# Functional significance of the electrocorticographic auditory responses in the premotor cortex

**DOI:** 10.3389/fnins.2015.00078

**Published:** 2015-03-16

**Authors:** Kazuyo Tanji, Kaori Sakurada, Hayato Funiu, Kenichiro Matsuda, Takamasa Kayama, Sayuri Ito, Kyoko Suzuki

**Affiliations:** ^1^Department of Clinical Neuroscience, Yamagata University Graduate School of MedicineYamagata, Japan; ^2^Department of Neurosurgery, Yamagata University Graduate School of MedicineYamagata, Japan

**Keywords:** premotor area, apraxia of speech, TMS, mirror neuron, motor theory of speech perception

## Abstract

Other than well-known motor activities in the precentral gyrus, functional magnetic resonance imaging (fMRI) studies have found that the ventral part of the precentral gyrus is activated in response to linguistic auditory stimuli. It has been proposed that the premotor cortex in the precentral gyrus is responsible for the comprehension of speech, but the precise function of this area is still debated because patients with frontal lesions that include the precentral gyrus do not exhibit disturbances in speech comprehension. We report on a patient who underwent resection of the tumor in the precentral gyrus with electrocorticographic recordings while she performed the verb generation task during awake brain craniotomy. Consistent with previous fMRI studies, high-gamma band auditory activity was observed in the precentral gyrus. Due to the location of the tumor, the patient underwent resection of the auditory responsive precentral area which resulted in the post-operative expression of a characteristic articulatory disturbance known as apraxia of speech (AOS). The language function of the patient was otherwise preserved and she exhibited intact comprehension of both spoken and written language. The present findings demonstrated that a lesion restricted to the ventral precentral gyrus is sufficient for the expression of AOS and suggest that the auditory-responsive area plays an important role in the execution of fluent speech rather than the comprehension of speech. These findings also confirm that the function of the premotor area is predominantly motor in nature and its sensory responses is more consistent with the “sensory theory of speech production,” in which it was proposed that sensory representations are used to guide motor-articulatory processes.

## Introduction

Other than well-known motor activities in the precentral gyrus, functional magnetic resonance imaging (fMRI) studies have found that the ventral part of the precentral gyrus is activated in response to linguistic auditory stimuli (Wilson et al., 2004). It has been suggested that this activity is best interpreted according to the “motor theory of speech perception” which argues that phonetic information is perceived in a “module,” or a biologically based link between perception and production specialized to detect the intended gestures of the speaker, rather than by translation from auditory impressions (Liberman and Mattingly, 1985). Accordingly, a number of recent studies have demonstrated that transcranial magnetic stimulation (TMS) to the left ventral premotor cortex modulates the efficiency and accuracy of phoneme comprehension. For example, deactivation of the left premotor cortex with repetitive TMS is associated with a decline in the accuracy of auditory syllable perception (Meister et al., 2007) while double TMS to the ventral precentral gyrus facilitates reaction time during speech perception (D'Ausilio et al., 2009). These results support the involvement of the motor cortices in phoneme comprehension which, in turn, underlies the motor theory of speech perception. However, it may not be possible to link these relatively minor TMS-induced behavioral effects with the symptoms of patients suffering from physical lesions in the precentral gyrus. Such TMS effects are reported to emerge only when the speech sounds are partially ambiguous with addition of noise and/or when the behavioral measure is reaction time rather than accuracy (Sato et al., 2009; Hickok et al., 2011). Additionally, clinical studies have clearly established that lesions in the precentral gyrus are associated with articulatory disturbances rather than speech comprehension disturbances (Duffy, 2012), which is more suggestive that the auditory response in the precentral gyrus can be interpreted with “sensory theory of speech production,” in which it was proposed that sensory representations are used to guide motor-articulatory processes, as the reverse relation of the one proposed in motor theories of speech perception (Venezia and Hickok, 2009; Hickok et al., 2011).

Cortical speech motor disorders are typically classified into two categories: dysarthria and apraxia of speech (AOS). Dysarthria is defined as speech disorders resulting from disturbances in muscular control of the speech mechanism (Darley et al., 1975). However, this term should be interpreted with caution because speech disturbances that result from cortical lesions are often broadly classified using the generic term “cortical dysarthria” and do not include a qualitative description of the speech disturbance (e.g., Kim et al., 2003). Patients with AOS could easily be misclassified as cortical dysarthria. AOS, also known as pure anarthria or aphemia, is characterized by unpredictable and irregular errors in the absence of paralytic disorders and linguistic disturbances. Although the original definition of AOS simply states that the condition is a motor planning or programming disturbance (Darley et al., 1975), the actual symptoms of AOS are not homogeneous, creating frequent disagreements in terms of diagnosis, even among experienced speech pathologists (Haley et al., 2012). It is likely that this debate continues because most patients do not display the pure symptoms of AOS (Laganaro, 2012), many cases present with comorbid AOS and dysarthria (Duffy, 2012), and, often, AOS is part of an aphasia syndrome.

A number of studies have attempted to better characterize the locations of lesions that accompany AOS (Dronkers, 1996; Hillis et al., 2004; Richardson et al., 2012). It has been proposed that lesioning of several structures, including the insula and Broca's area, is crucial for development of AOS. However, the exact nature of the relationship between lesion location and clinical symptoms has yet to be established. This may be due to the fact that pure cases of AOS are rare and that most data on this disorder are based on findings from cases with large lesions in which AOS manifests as a component of an aphasia syndrome. Although some researchers have argued that damage to Broca's area is crucial for development of AOS (Hillis et al., 2004), this notion is not consistent with the fact that lesions restricted to Broca's area do not result in AOS (Mohr et al., 1978). Moreover, such studies typically include patients with large lesions that span the prefrontal cortex, the precentral gyrus, and the underlying white matter (Hillis et al., 2004).

On the other hand, AOS has consistently been associated with lesions that are restricted to the precentral gyrus. The first such case that included a sufficient description of both the location of the lesion and the qualities of the articulatory disturbance was reported by Lecours and Lhermitte (1976). These authors found that phonetic disintegration syndrome, which is disorganization of the exclusive choice of a certain number of features and their largely cotemporal integration into a more complex unit (the phoneme), resulted from a lesion restricted to the ventral precentral gyrus. This report was followed by several studies describing lesions that were generally restricted to the precentral gyrus but that exhibited heterogeneous clinical symptoms. Although a majority of these cases featured a phonetic disorder (such as a distortion in speech), there were also cases in which precentral lesions led to phonemic issues with sequential errors (Sasanuma, 1971; Tanji et al., 2001), cases that were characterized by phonemic issues without sequential errors (Larner et al., 2004), and cases that were characterized by a foreign accent syndrome (Sakurai et al., 2014). It is notable that the clinical symptoms associated with lesions in the precentral gyrus, which tends to be regarded as a structure supporting relatively simple motor functions, are so diverse. Based on the findings of these case studies, it is clear that the exact relationship between a lesion and the symptoms thereof in terms of articulatory disturbances, even within the precentral gyrus, has yet to be established. Such heterogeneity is likely the result of not only variability in the location of the lesioned area but also variations of functional organization within the precentral gyrus.

In fact, organization within the precentral gyrus is not as simple as is generally believed. The term “precentral gyrus” is often used as a synonym for the primary motor area, or Brodmann area (BA) 4, and the fact that the convex surface thereof is mostly occupied by the premotor cortex, or BA 6 (Rizzolatti et al., 1998), is often overlooked. Depending on its precise location within the precentral gyrus, a lesion could affect BA 4 and BA 6 to varying degrees and this may explain the co-occurrence of dysarthria and AOS. Additionally, it has been proposed that dissociation of premotor function occurs along the dorsoventral axis (Duffau, 2003). Given the complexities of the articulatory disorders that result from precentral lesions, it is possible that other unknown organizing principles also influence the functional distribution of premotor activity.

Neuroimaging studies affording excellent temporal and spatial resolution should help reveal the nature of such organizing principles and elucidate their functional distribution within the precentral gyrus. Thus, the present study details the electrophysiological responses induced by a verb generation task in the precentral premotor areas of a patient undergoing an awake craniotomy for tumor resection. The present study used electrocorticography (ECoG) in an attempt to confirm the reproducibility of the auditory activity that has been previously reported by fMRI studies (Wilson et al., 2004). It was expected that the distribution of the auditory activity within the precentral gyrus in response to linguistic auditory stimuli would be revealed, due to the excellent temporal and spatial resolution provided by direct cortical recording. To this end, the present study used equivalent linguistic stimuli in the form of written words (a visual modality) to confirm whether the activity was modality-specific or cross-modal; to the best of our knowledge, this has never been assessed in humans. Additionally, a description of the articulatory disorder in this patient that developed following the resection of this area of the precentral gyrus is provided.

## Materials and methods

### Subject

**Figure 1:**
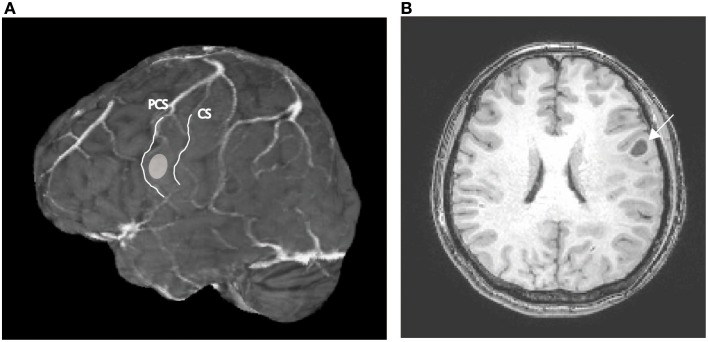
**(A)** Preoperative MR image of the patient's brain; the ellipsoid indicates the location of the tumor. CS, central sulcus; PCS, precentral sulcus. **(B)** Axial section of the preoperative T1-weighted MRI; the arrow indicates the tumor in the left precentral gyrus.

A 52 year-old right-handed woman was admitted to a hospital after an episode of partial seizure with secondary generalization. Magnetic resonance imaging revealed a brain tumor in the left precentral gyrus (Figure 1), and was referred to our hospital for surgical consultation. She underwent an awake craniotomy for resection of the tumor and, at the same time, participated in this language mapping experiment. This study was approved by the ethics committee of the Faculty of Medicine at Yamagata University and posed no additional risk to the patient. The patient provided signed informed consent after a detailed explanation of the procedure. She did not have any preoperative disturbance in language or motor function, except for very mild dysarthria. The Wechsler Adult Intelligence Scale-III (WAIS-III) revealed a verbal intelligence quotient (IQ) of 96, a performance IQ of 95, and a full-scale IQ of 95. The patient was initially anesthetized with propofol but all medication was discontinued prior to the start of the experiment.

### Stimuli and tasks

The task began when a fixation cross appeared at the center of a screen. Subsequently, a series of nouns was presented and the patient was asked to think of an associated verb for each noun and then make an overt response after the “Go” signal was visually displayed 2 s after presentation of the nouns (delayed response design). The nouns were presented either visually or auditorily in separate trial runs. In the first run, the patient was asked to only listen to auditorily presented nouns. In the remainder of the runs, the patient was asked to make an overt response to each of the nouns that were presented auditorily in the second and fourth runs, and visually in the third and fifth runs after the “Go” signal. The delayed response design was intended to separate and differentiate the neural activities that were related to sensory stimulus processing, motor processing, and the delay period. Each run consisted of the presentation of 33 words and, thus, a total of 66 words were presented for the verb generation tasks in each modality.

**Figure 2:**
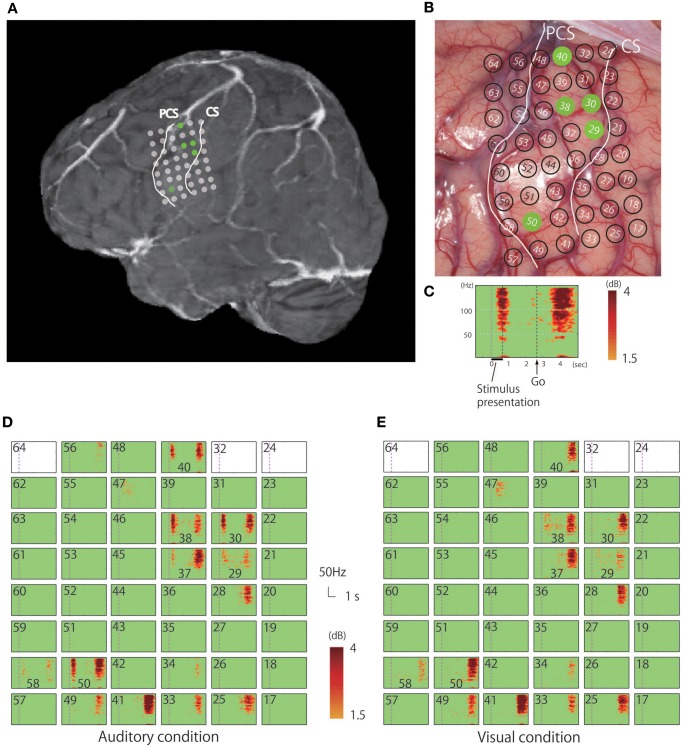
**(A)** Rendered MR image in which the dots indicate the positions of the electrodes from which responses to the verb generation task were recorded. **(B)** Positioning of electrodes overlaid on the cortical surface prior to tumor resection; green dots indicate electrodes in the precentral gyrus that recorded auditory responses. **(C)** Magnified view of a representative time-frequency plot (channel 50). The horizontal axis represents time, the vertical axis represents frequency, and the colors indicate the magnitudes of the response (dB) relative to the baseline period; the purple dotted line indicates the time (0 s) when the noun was presented. **(D)** Time-frequency plots of the recorded signal in response to the verb generation task with auditory noun presentation. **(E)** Time-frequency plots of the recorded signal in response to the verb generation task with visual noun presentation. Blank rectangles indicate bad channels.

While in the operating room, all auditory stimuli were presented at a comfortable sound level via a loudspeaker placed 1 m away from the patient, and all visual stimuli were presented in the center of a computer monitor (refresh rate: 60 Hz) placed 0.8 m from the patient; all nouns were presented with an inter-stimulus interval of 6 s. Only concrete high-familiarity nouns were used for this task and the patient was familiarized with the procedure prior to her operation. The intraoperative performance of the patient was 92.4% for the auditory task and 97.0% for the visual task. The electrocorticogram was recorded from electrodes that were temporarily placed directly on and surrounding the precentral gyrus, with an inter-electrode distance of 5 mm (Figures 2A,B). Only signals from electrodes that covered the pericentral gyri were analyzed in the present study.

#### Data analysis

The potentials at each electrode were re-referenced to an intracranial average electrode. The signals were digitized at 1000 Hz, recorded onto a computer hard disk, and the data bandpass-filtered at 0.1–300 Hz. Any trial containing an epileptic discharge or other artifact was eliminated from further analysis. Event-related spectral perturbation (ERSP) measures the average dynamic changes in the amplitude of the broadband electroencephalographic frequency spectrum as a function of time relative to an experimental event (Makeig, 1993). In the present study, time-frequency analyses were conducted using three-cycle standard Morlet wavelets at each frequency from 2.9 to 150 Hz progressing through 6.5 s data epochs (from 1.5 s prior to and 5 s after the stimulus onset) in 26.8 ms steps. A bootstrap resampling method was used to test whether the ERSP deviations in spectral power in the post-stimulus interval were significantly larger than in the pre-stimulus period. Bootstrapping addresses the significance of deviations from pre-stimulus baseline power by randomly resampling the spectral estimates of the selected pre-stimulus epoch data of each trial and then averaging these, thus constructing a surrogate baseline data distribution. Additionally, the False Discovery Rate (FDR) algorithm was applied to correct for multiple comparisons. The ERSP plots provided time-frequency points at which the mean log power was significantly higher or lower (bootstrap, *p* < 0.05, FDR-corrected) than the mean power during the 1.5 s pre-stimulus baseline period for the same epochs. The increase in power in a given electrode was considered to be significant if a cluster of pixels of power larger than 1.5 dB in the gamma band range was arranged in a continuous array spanning at least 40 Hz in width. The clusters that fulfilled this condition were detected with the Matlab function bwconncomp.m, which was used for finding connected components in an image.

#### Speech analysis (post-operative)

In the present case study, resection of the ventral portion of the precentral gyrus was inevitable due to the following clinical findings: (1) other than the pathological Gadolinium-enhancement on preoperative MRI, which is associated with more aggressive lesions (Upadhyay and Waldman, 2011), thallium uptake was high on the preoperative single photon emission computed tomography (SPECT), which is known to be a good indicator of the histological grade (Comte et al., 2006). (2) Histological diagnosis of glioblastoma multiforme was made on the intraoperative as well as post-operative histological examinations. Her post-operative speech findings were first evaluated 1 month after the surgery with The Japanese Standard Language Test of Aphasia (SLTA), which is the standardized test battery most commonly used to evaluate Japanese aphasic patients. This test consists of 26 subtests addressing four modalities (speaking, listening, writing, and reading), three component levels (phoneme, word, and sentence), and two character types of Japanese language: kanji (morphograms) and kana (syllabograms). Because the patient developed non-aphasic speech disturbances post-operatively, the qualitative features of her articulatory disturbance were recorded on an IC-recorder and analyzed. One month after her surgery, the articulation of the patient was evaluated at the word level using the same list of 180 words with 3–5 syllables to examine her repetition and reading aloud abilities, on the same day. The error types were classified as follows: sequential phonemic error, omission (of the word-initial consonant), non-sequential phonemic error, and distortion. Sequential errors were defined as errors caused by the inadequate ordering of phonemes or syllables in which the distance between the original position of a target and its actual position was within two syllables. As described previously (LaPointe and Horner, 1976) these errors were subdivided as follows: pre-positioning, in which a phoneme is replaced by one that occurs later in the word; post-positioning, in which a phoneme is replaced by one that occurs earlier in the word; and metathesis, in which two phonemes switch places. Classification as a non-sequential phonemic error was made conservatively, such that a phoneme was considered to fit into the distortion category if it shared the place of articulation with the target syllable even when it was judged to be free of distortion. Two experienced neurologists and an experienced speech pathologist transcribed the speech sample of the patient. First, one examiner orthographically transcribed the speech sample and the resulting transcript was then independently verified or modified by the other two examiners, yielding a total of three transcripts. These three transcripts were then compared and consolidated to produce a composite transcript that reflected the consensus of at least two of the three examiners. No further analyses were performed on utterances for which no agreement could be reached. If the patient discontinued articulating a word halfway through her verbalization thereof, the errors in the discontinued word were evaluated if it was intelligible and decipherable from the target word, or by her corrected utterance.

## Results

### ECoG findings from the verb generation task

**Figure 3:**
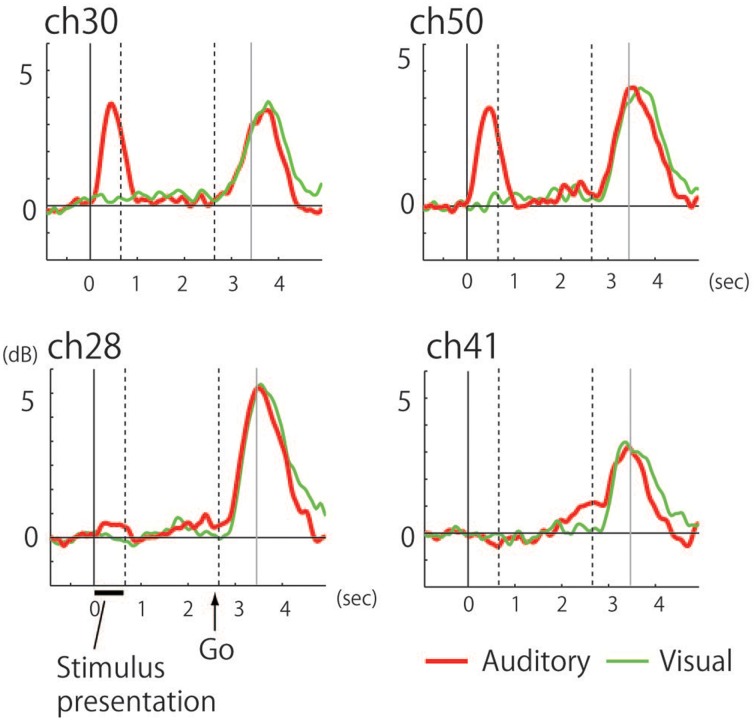
**Power curve in the 60–140 Hz band under auditory (red) and visual (green) conditions**. Indicating **(upper row)** biphasic responses with the auditory-selective response accompanied by the motor response (Channels 30 and 50) and **(lower row)** monophasic responses with purely motor responses (Channels 28 and 41). The end of stimulus presentation is indicated by the dotted line at 0.65 s while the other dotted line indicates the timing of the “Go” signal at 2.65 s. Gray line indicates mean voice onset time of the subject's response.

The recorded area was localized based on the central sulcus and the precentral sulcus which were identified according to the hand-knob sign (Yousry et al., 1997), and preoperative fMRI was used to locate the hand motor area (thumb opposition task). Time-frequency analysis of the electrocorticogram revealed significant responses characterized by a broadband high-gamma band in multiple electrodes on the precentral gyrus (Figure 2C). Whereas early phase responses that were time-locked to the stimulus (noun) presentation were observed under auditory conditions, these responses were not observed following visual presentation of stimuli (Figures 2D,E, 3). Five electrodes in the precentral gyrus exhibited activity in response to auditory stimuli (Channels 29, 30, 38, 40, and 50; Figure 2D). In contrast, late-phase responses that were time-locked to the “Go” signal were observed in common electrodes (Figures 2D,E) that represented the motor response. All of the sites exhibiting auditory responses were characterized by biphasic activity, with early and late phases (e.g., Channels 30 and 50; Figure 3). Also, some electrodes exhibited only late-phase activity that did not accompany any sensory activity (e.g., Channels 28 and 41; Figure 3), and likely corresponded to a purely motor area. Passive listening to auditory presentation of the noun (without a verbal response) induced significant responses from the set of electrodes that exhibited early-phase responses in the auditory verb generation task (Supplementary Figure 1). It is of note that adjacent electrodes often displayed completely different response patterns (e.g., Channel 50 vs. Channel 41; Figure 2D).

### Post-operative speech findings

**Figure 4:**
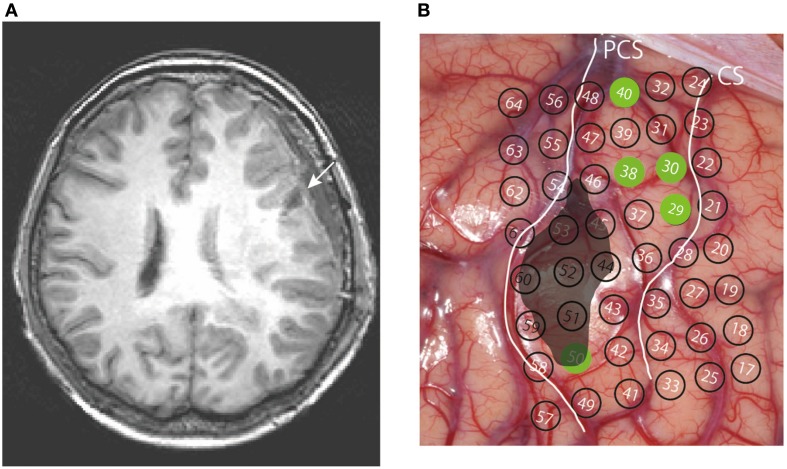
**(A)** Post-operative axial MR image of the resected region (arrow) in a slice most similar to Figure 1B. **(B)** Resected area (black) superimposed on the cortical surface, with the positioning of the electrodes.

In the present case study, the ventral portion of the precentral area, which included an auditory-responsive cluster, had to be resected due to the location of the tumor. Excision of the tumor was done with minimal damage to surrounding structures with the extent of resection (EOR) of 100% (Figure 4). Post-operatively, the patient suffered from AOS. She did not show any apparent orofacial motor weakness or buccofacial apraxia and her language function, including auditory and reading comprehension of syllables, words, and sentences, and the writing of words and sentences, was normal according to the SLTA: the patient scored full (10 out of 10 questions) in all of the following 9 subscores of the SLTA which were relevant for the present evaluation, namely: “auditory comprehension of words,” “auditory comprehension of short sentences,” “auditory comprehension (to obey verbal commands),” “auditory comprehension of syllables,” “written naming of pictures with kanji letters,” “written naming of pictures with kana letters,” “dictation of kana letters,” “writing to dictation with kanji words,” “writing to dictation of kana words.” Her speech was characterized by frequent distortion, a slow overall rate with abnormal prosody, lengthened segment durations and segmentation, and variable articulatory disturbances including sound distortions, substitutions, omissions, and sequential errors. Some of the errors were interpreted to be phonemic rather than phonetic based on their sequential nature (Table 1). In the word-level reading and repetition task, sequential errors were identified in 15.8% of words (15.6% during repetition and 16.1% during reading). Of the 57 sequential errors, 53 (93%) were pre-positioning, three (5.6%) were post-positioning, and one was equivocal. Metathesis was not observed. Omissions of the word-initial consonant were identified in 8.0% of the words, non-sequential phonemic errors in 13.3% of the words, and distortion in 21.7% of the words. Additional characteristic finding was that when the patient had trouble pronouncing the syllable “mu” and was not successful even after several trials; subsequently, she would begin with the syllable “ma” and say “ma mi mu,” which is a part of the M row of the “50-on,” an overlearned Japanese kana syllabary. The patient strategically used the 50-on syllabary as a cue and eventually could successfully pronounce “mu.” Similar behavior was observed six times during the session.

**Table 1:** **Examples of sequential errors**.

**Stimulus word**	**Subject response**	
Tansu (closet)	“sansu”	(pre-positioning of “s” of “su”)
Sutanpu (stamp)	“supanpu”	(pre-positioning of “p” of “pu”)
Nukamiso (bean paste)	“ni… nukamiso”	(pre-positioning of “i” of “mi”)
Hikidashi (drawer)	“ki… hikidashi”	(pre-positioning of “ki”)
Manaita (cutting board)	“mata… manaita”	(pre-positioning of “ta”)
Omusubi (rice ball)	“omusubu”	(post-positioning of “u” of “su”)

## Discussion

In the present study, we recorded ECoG from the pericentral region of a patient with a brain tumor in this area during an awake craniotomy. Activities were observed in the precentral gyrus in response to auditory, but not visual, stimuli, which suggests that the observed responses did not reflect general linguistic processes, such as word production or comprehension, or the premotor preparatory activity that precedes articulation, because no activities were observed in response to the visual presentation of nouns in the same task. Resection of a part of the ventral precentral area, from which a positive ECoG auditory response was detected (Channel 50), was inevitable due to the location of the tumor. Consistent with previous case studies of patients with lesions in the precentral gyrus, the present patient did not develop any post-operative comprehension disorders. However, she did develop AOS which was characterized by dysprosodic, slow, irregular speech with distortion and phoneme substitutions, including multiple sequential errors (pre-positioning and post-positioning). This was likely because the lesion included the auditory-responsive precentral premotor area.

### Role of the auditory-responsive ventral premotor subarea in speech production

In clinical studies, the uniformity of functional structure within the precentral gyrus has rarely been questioned, except for the distinction between BA 4 and BA 6. In experimental studies using monkeys, the ventral premotor area (PMv) is thought to specialize in direct sensory-motor mapping while the dorsal premotor area (PMd) is thought to be involved in indirect sensory-motor mapping (Hoshi and Tanji, 2004). PMv in monkeys has been extensively discussed as a structure associated with mirror neurons, which fire during the execution of an action as well as during the observation of an action, and are proposed to support the understanding of others' actions via motor simulation (Di Pellegrino et al., 1992). A large number of neuroimaging studies in humans have demonstrated the existence of the mirror mechanism in humans in the posterior inferior frontal area and the PMv (Rizzolatti and Craighero, 2004). Some see BA44 as the human homolog of macaque F5 (Rizzolatti and Arbib, 1998), but others see BA6 as the human homolog of F5 (Morin and Grèzes, 2008). Mirror neuron findings were generalized to speech understanding (Rizzolatti and Arbib, 1998) based on analogy to the motor theory of speech perception (Liberman and Mattingly, 1985). However, as was mentioned in Introduction, it is increasingly clear that motor system could be crucially recruited for speech perception only under certain conditions that make speech discrimination hard (D'Ausilio et al., 2012; Krieger-Redwood et al., 2013). Consistent with these recent findings, our patient performed flawless on all standard tasks evaluating language comprehension including phoneme identification task. Although only parts of the auditory-responsive area have been resected and it is possible that the remaining regions could account for intact speech perception in this case, previously reported cases with lesions restricted to precentral gyrus also showed intact phoneme identification in the same sets of tasks (Tanji et al., 2001; Kasahata, 2013). Also, if premotor neurons are responsible for understanding of a certain behavior, it would be expected that the auditory response accompanied by this understanding should be cross-modal, as was proposed following the discovery of audiovisual mirror neurons in monkeys (Kohler et al., 2002). However, in the present study, the dissociation between the neural activities induced by auditorily and visually presented words suggests that this process was sensory-specific; the auditory modality, in this case. Taken together, the auditory-motor response observed in our case is not likely to be interpreted as having a proposed “mirror” property.

The auditory-motor activation of the PMv in our case might be explained better with recently proposed model of language processing proposing two parallel streams, in which dorsal stream serves auditory–motor integration (Hickok and Poeppel, 2007). The dorsal auditory stream was found to be closely linked with the vocal motor control system, and PMv was proposed to serve as a key component in the theories that proposes production-oriented auditory processing of the dorsal stream (Houde and Nagarajan, 2011; Guenther and Vladusich, 2012). DIVA (Directions Into Velocities of Articulators) model, a neural model of speech acquisition and production that provides a conceptual and computational framework for interpreting data concerning brain activity during speech and language task (Guenther et al., 2006), proposed that speech sound map neurons gradually acquire the feedforward motor command programs corresponding to the auditory target sounds. The link between perception and action thus arises in the DIVA model because the motor reference frame is brought into register with the auditory reference frame. The model therefore predicts a causal relationship between the speech sounds acquired in auditory coordinates and their associated motor programs (Guenther and Vladusich, 2012). Consistent with the DIVA model, the state feedback control model (SFC) proposes that auditory input is not only used for comprehension during listening, but also for speech production. In this model physical auditory feedback of one's own speech is compared with a prediction derived from efference copy of the motor output, and used to train and update the internal model. It was postulated that the auditory-responsive portion of the ventral premotor area is ideally placed to serve this intermediary role that mediates the prediction and correction processes running between motor and sensory cortices (Houde and Nagarajan, 2011). The fact that PMv is active during passive listening to speech is consistent with the reciprocal connections between premotor and sensory areas and was regarded as the evidence for premotor cortex playing such an intermediary role in speech production.

A recent study investigated ECoG responses in the premotor area during the articulation of pseudo-words under two conditions: one in which the subjects were required to repeat the presented syllables and one in which the subjects were required to respond using pre-associated pseudo-words (Cogan et al., 2014). Under both conditions, the decoding of the initial auditory response in the premotor cortex successfully predicted the paired response, which is consistent with the existence of the representation of “parity” between sensory and motor processes in the premotor cortex.

### Implication for the pathophysiology of AOS

A recent study investigating the lesion distributions of AOS patients without concomitant aphasia found that the lesions were predominantly located in the left premotor cortex (BA 6) (Jacks et al., 2010). Furthermore, single-case studies on patients with lesions contained within the precentral gyrus have repeatedly reported the development of AOS (e.g., Lecours and Lhermitte, 1976). Along with these case studies, the present findings confirm that complex speech disturbances characterized by prosodic abnormalities, inconsistent distortion, and phonemic inaccuracies with sequential errors, result from lesions in the precentral gyrus.

Several issues render the diagnosis of AOS difficult, and indeed debatable (see Introduction), such as the problem of whether articulatory disturbances should be classified as purely phonetic instead of phonemic. While AOS is predominantly characterized by distorted sounds not attributable to deficits in muscle tone or reflexes, which would reflect disturbances during the encoding of phonological patterns into appropriate speech movements (Canter et al., 1985), it is argued that speech disturbances accompanied by good acoustically and perceptually produced sounds that are missequenced should be classified as phonemic paraphasias, which are typically associated with posterior lesions (McNeil et al., 2009). In fact, it has been asserted that the debate over whether AOS is a phonological disorder or a motor programming disorder is fatuous because, by definition, AOS is a motor planning/programing disorder (McNeil et al., 2009). The clear-cut demarcation of an abstract amodal phonology from the motor mechanisms of speaking support these arguments (Ziegler et al., 2012). However, it is challenging to delineate a clear boundary between AOS and phonological paraphasias based on the distinction between phonemic and phonetic errors because patients with AOS often produce apparently well-articulated phonemic errors (Goodglass, 1993; Ziegler et al., 2012), as in the present case. Lapointe and Johns found that all 13 of their AOS patients produced sequential errors, albeit in various proportions from 0.8 to 20% of all errors (La Pointe and Johns, 1975). In other studies, extreme cases of AOS have been reported. One such patient with a frontal lesion that included the precentral gyrus was primarily characterized by metathesis in which approximately 90% of the phonemic errors were classified as sequential errors (Sasanuma, 1971). An MRI that was performed later revealed a cortical lesion that involved the left precentral gyrus and the insula and extended to the underlying subcortical regions (Kawachi, 1987). Another similar case with a lesion that was restricted to the precentral gyrus and insula developed predominantly sequential errors (52% of all phonemic errors) that were typically due to pre-positioning (Tanji et al., 2001). Lesions common to both cases involved the ventral precentral gyrus and the insula. These authors suggested that these regions may be responsible for articulatory sequencing during which the proper timing for each phoneme and syllable are coded (Tanji et al., 2001). In the present case, sequential errors of phonemes or syllables were identified in 16% of the words that were repeated or read aloud. Although it remains uncertain whether or not non-sequential phonemic errors result from random distortion, sequential errors can be confidently identified as phonemic because these types of errors reflect the influence of contextual phonemic information. Although it has been argued that sequential errors result exclusively from disturbances in the phonological system of the posterior language area (McNeil et al., 2009), cases such as these indicate that sequential errors can also be caused by lesions localized to the premotor area. It has been suggested that the primitives of speech motor programs are the size of syllables and the overlearned syllable-sized programs form a mental syllabary that is stored in the ventral premotor cortex (Levelt, 2001). The cited authors further proposed that word forms comprised of two or more syllables are not stored as pre-specified motor routines and, consequently, when a word is articulated, pre-compiled programs should assemble into sequence. Hence, this type of sequencing of syllables into larger utterances must be performed online during speech production and should depend on the motor execution system, including the premotor cortex. The mechanism to “bind” the temporally segregated sub-entities of these events into a single unified entity was proposed to be provided by the PMv (Fiebach and Schubotz, 2006). This is also consistent with the existence of the motor phonological system (Hickok et al., 2011), which implies that the premotor cortex is involved in the phonological process.

Another finding from the present study that supports the division of labor between the sensory phonological and motor phonological processes is the characteristic groping behavior regarding the articulation of “mu” (see Results). The most likely interpretation of this behavior is that the patient in the present study had a clear idea of the target syllable in terms of sensory phonology, which is supported by the fact that she was able to write the letter correctly, but she was not able to retrieve an articulatory motor counterpart therefor. However, she was able to overcome this issue using the “50-on,” an overlearned Japanese kana-syllabary, as a contextual cue. This is consistent with the well-accepted characterization of AOS: The affected patient cannot speak properly although he or she is aware of what he or she wants to say and how it should sound (Hillis et al., 2004). As in the present case, some of the struggling behavior that is often observed in patients with AOS may be interpreted as an inability to recall the motor plan for production of a speech sound (Van der Merwe, 2009).

Turning to the heterogeneity of AOS symptoms, if each patient with AOS is carefully assessed on a case-by-case basis, even patients with circumscribed lesions in the ventral precentral cortex can show a diversity of symptoms (Kasahata, 2013). This likely reflects the heterogeneity of physiological properties within the precentral gyrus. As indicated by the ECoG findings in the present study, subregions with distinct functional properties exist within circumscribed areas of the precentral cortex. It is reasonable to assume that a slight shift in the location of a lesion would thus result in different symptoms. As discussed above, resection of the tumor in the present study occurred in a cortical area associated with characteristic sensory-motor responses. To the best of our knowledge, this is the first patient study which has reported AOS as a consequence of the resection of a brain region within the precentral gyrus, with a confirmed auditory response.

## Conclusions

Based on the dual-route model (Hickok and Poeppel, 2007), premotor auditory activity likely reflects input from the area Spt at the parietal-temporal boundary via the arcuate fasciculus. Moreover, recent ECoG data suggest that the area Spt exhibits premotor activity which precedes the production of speech (Edwards et al., 2010). This finding is consistent with the clinical observation that a lesion in the temporoparietal junction including the area Spt can result in phonemic paraphasia (Damasio and Damasio, 1980). The present observation that a lesion in the premotor subregion of the precentral gyrus, which processes auditory inputs, results in AOS, suggests that the premotor area plays an important role during articulation, using input from the auditory linguistic system. These findings also support the argument that the premotor area is more appropriately interpreted using the “sensory theory of speech production” rather than the “motor theory of speech perception.”

### Conflict of interest statement

The authors declare that the research was conducted in the absence of any commercial or financial relationships that could be construed as a potential conflict of interest.

## Supplementary material

Supplementary Figure 1 **Passive listening to auditorily presented nouns induced significant responses in electrodes common to the verb generation condition, that were timelocked to the auditory stimuli (Channels 29, 30, 38, 40, and 50)**.
