# Supplementary figures and images for: Functional significance of the electrocorticographic auditory responses in the premotor cortex
Source: Front Neurosci. 2015 Mar 16;9:78. doi: 10.3389/fnins.2015.00078 (PMC4360713; doi:10.3389/fnins.2015.00078)

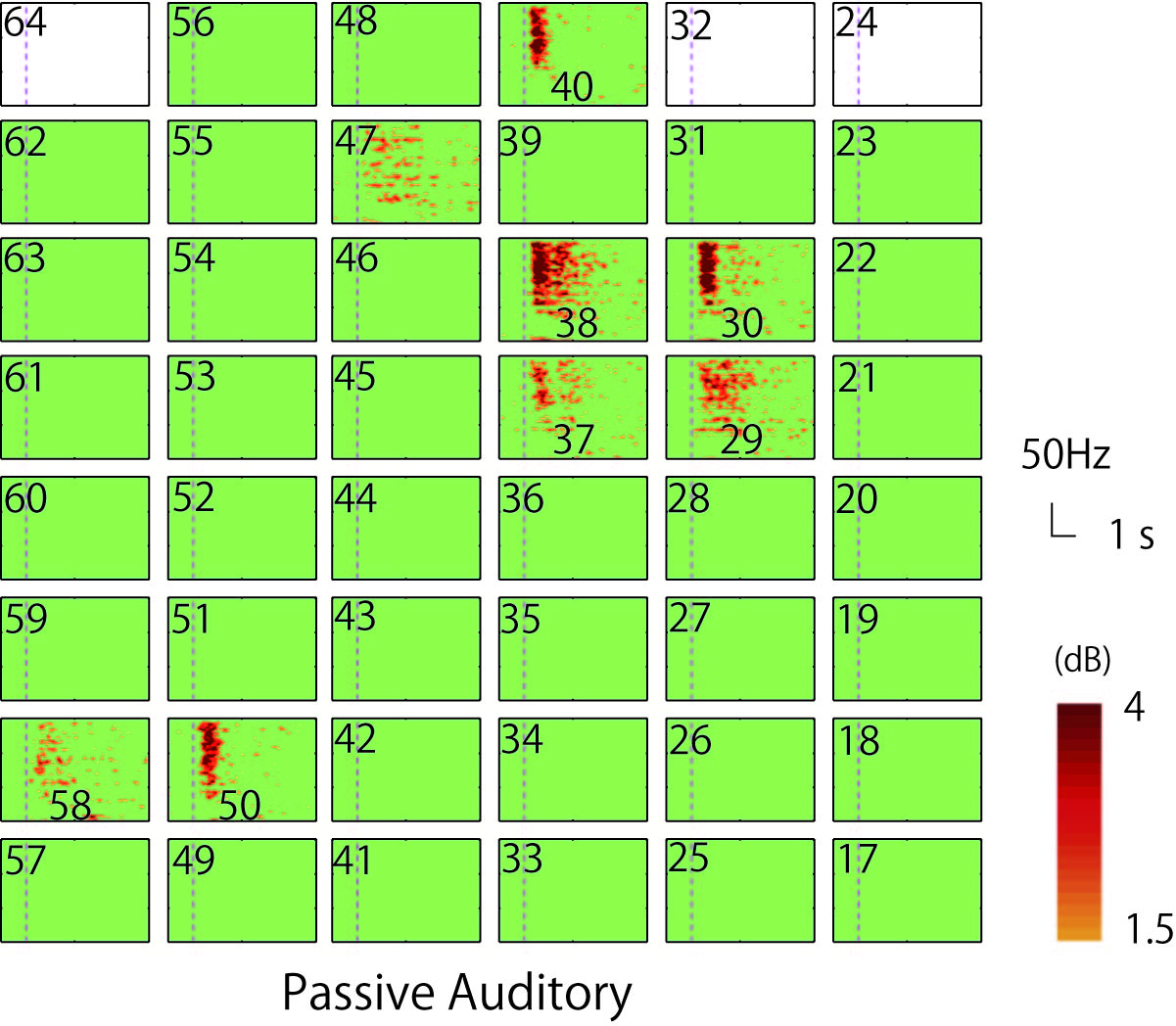

Supplement: Supplementary Figure 1 — Passive listening to auditorily presented nouns induced significant responses in electrodes common to the verb generation condition, that were timelocked to the auditory stimuli (Channels 29, 30, 38, 40, and 50). [file Image1.JPEG]
